# Human Pegivirus Type 1 Coinfections: Epidemiology, Heterogeneity and Clinical Implications

**DOI:** 10.1099/jgv.0.002365

**Published:** 2026-11-05

**Authors:** Mathieu Garand, Ching Ching Zhang, Grace Guo, Ting Zhong, Jack T. Stapleton, Pirooz Eghtesady

**Affiliations:** 1 Division of Pediatric Cardiothoracic Surgery, Department of Surgery, Washington University School of Medicine, St. Louis, Missouri, USA; 2 Division of Infectious Diseases, Departments of Internal Medicine, Microbiology and Immunology, University of Iowa and the Iowa City Veterans Administration Medical Center, Iowa City, USA

**Keywords:** GBV-C, *Hepaciviridae*, Human Pegivirus, narrative review, polymicrobial infections, population prevalence, viruses, viral coinfection

## Abstract

This narrative review explores the complex landscape of coinfections involving Human Pegivirus Type 1 (HPgV), a member of the *Hepaciviridae* family previously known as GB virus type C or hepatitis G virus. Structurally similar to hepatitis C virus (HCV), HPgV is lymphotropic and while viraemia in healthy donors is asymptomatic, it has been associated with the onset of several non-Hodgkin lymphoma subtypes. We begin with an overview of HPgV’s genomic features, classification, transmission routes and natural history, including spontaneous clearance and post-infection anti-E2 antibody development. As of 03 February 2026, a PubMed search yielded 346 relevant studies from which data were synthesized, reporting HPgV coinfection across human immunodeficiency virus (HIV), HCV, hepatitis B virus and other viral diseases – highlighting genotype distributions, geographic variability and population-specific patterns. In summary, HPgV shows a reproducible signal in HIV coinfections, suggesting potential therapeutic benefits, while its impact on chronic hepatitis, acute infections and lymphoma risk remains preliminary and requires further investigation. This review highlights the need for rigorous prospective studies with standardized protocols to clarify HPgV’s true impact on human health and its potential for therapeutic application.

## Data Availability

All data supporting the findings of this review will be made available online in accordance with the journal’s data-sharing and accessibility policies.

## Introduction

Belonging to the genus Pegivirus of the *Hepaciviridae*, Human Pegivirus Type 1 (HPgV-1) is an enveloped RNA virus estimated to be 50 nm in diameter [1]. Its viral genome structure is most similar to hepatitis C virus (HCV), both containing a 9.4 kb positive-sense ssRNA genome, 5′- and 3′-UTRs, and an ORF encoding a single polyprotein cleaved by viral and cellular proteases to produce functional and structural proteins, respectively [2, 3]. Two known species currently exist within the Pegivirus H species, HPgV-1 and HPgV-2. HPgV-1 is more prevalent in humans and will be the focus of this review. HPgV-2 is associated with HCV coinfection and has an additional protein (the X protein) in the structural coding region compared to HPgV-1. HPgV-1 (HPgV going forward) was initially named GB virus type C (GBV-C) or hepatitis G virus (HGV) by two different groups. Diseases associated with GB type viruses were first identified in 1967 in marmosets, and the etiologic agent was identified in 1995 when two different RNA viruses were isolated from the marmoset serum [4]. These were called GB virus-A and -B. A related virus was found in humans that was called GBV-C [4]. Another group identified a similar virus in a patient with chronic hepatitis and called the virus HGV. Subsequent molecular characterization showed GBV-C and HGV are strains of the same species, later named Pegivirus [5] with the ‘pe’ nomenclature in Pegivirus originating from observed persistent infections. Given the host species is limited to humans, it was officially named Human Pegivirus (HPgV) in 2013 [6].

HPgV infection, defined as the presence of HPgV RNA, is characterized by frequent spontaneous viral clearance with ~80% of immunocompetent individuals clearing viraemia within 2 years of acquisition [7, 8]. This pattern places HPgV between acute adult hepatitis B virus (HBV) infection – which clears in the majority of adults (>90% [9]), typically within months – and HCV where spontaneous clearance occurs in a minority of cases (<29% [10]) and predominantly within the first 6 months after infection. Unlike HBV and HCV, HPgV clearance is often delayed and occurs in the absence of a robust or durable antibody response, underscoring its distinct interaction with host immunity. For the other 20%, persistent infection could be due to the high concentration of lipids that mask HPgV neutralization epitopes [5] or the reduction of T-cell activation via T-cell receptor (TCR) due to HPgV’s major envelope glycoprotein (E2) [11]. After clearing HPgV viraemia, antibodies to the E2 protein are detected although they appear to be partially, rather than completely, protective against reinfection [12, 13]. To date, there have been no experiments to directly show that the anti-E2 antibody limits infectivity due to the lack of an *in vitro* replication system for HPgV. Less than 10% of all HPgV viraemic individuals develop a detectable coexistent anti-E2 antibody [7, 14, 15]. The exact reasoning for the lack of antibodies is unknown, but it‘s possible that HPgV has an immunosuppressive ability through its E2 protein, or the low-level production of virus per cell leads to reduced innate and, thus, adaptive immunity. HPgV persistence is widely observed in the context of immunosuppression or coinfections [16–19]. In the literature, ‘persistence’ can be defined in two ways: 1) continued detection of the same viral strain in the blood over an extended period or 2) repeated detection of HPgV viraemia over time. Only a limited number of longitudinal studies have performed sequencing of the Pegivirus genome and confirmed reinfection of the former strain [20, 21]. A 1999 study of a cohort including children treated for haematological malignancy reported HPgV clearance during the off-therapy period where immunocompetence seemed to be restored, and subsequent reactivation of the same strain during the second remission induction where the patient becomes immunocompromised again [20]. Furthermore, there is no comparable longitudinal data for healthy individuals, as frequent blood drawing and viraemia monitoring are rarely performed in this population. These limitations should be considered when interpreting reported rates of persistence which are best understood in a disease-specific context.

Historically, HPgV infections have been presumed to be benign and hence, lack of surveillance in the clinical setting. More recently, however, a few studies have reported associations between HPgV infection and non-Hodgkin lymphomas (NHLs) [22–25], encephalomyelitis in immunosuppressed patients [26], and Parkinson’s disease [27] although causal relationships have not been established. The association with NHL observed with most major NHL subtypes (but not small lymphocytic lymphoma and its leukaemic counterpart and chronic lymphocytic leukaemia) suggests lymphotropism (particularly T cells), potentially through impaired immune surveillance [2, 3, 28]. Three North American case-control studies strengthen this link: HPgV RNA was detected more often in NHL than in controls – 4.5 vs 1.8% [22], 1.8 vs 0.5% [23] and 10.1 vs 5% [24] – with the highest odds observed in plasma from patients with diffuse large B-cell lymphoma. Lower HPgV viral loads in NHL cases [23] and elevated cytokines such as IL-2R, MIP-1α and CXCL9 in HPgV-positive patients [24] point to chronic, immune-modulating infection as a potential driver. A commentary by Stapleton *et al*. highlights plausible biological mechanisms (e.g. altered CD4, Fas, chemokine signalling) but notes geographic heterogeneity and calls for larger, prospective cohorts and standardized antibody detection assays to confirm causality [29].

The known methods of transmission of HPgV include blood exposure (e.g. transfusion), sexual exposure, intravenous drug use (IDU) and, less often, mother to child. In developed countries, the weighted average of HPgV viraemia prevalence among healthy blood donors is 2.3% among 27 studies [interquartile range (IQR): 1.0–3.4%] [7, 14, 30–53]; prevalence is 4.2% in the USA [48]. The prevalence of HPgV across all studies is higher in those who are also infected with another bloodborne or sexually transmitted infection or have undergone organ transplantation compared to healthy blood donors. For example, one study found that among human immunodeficiency virus (HIV)-infected patients, each additional unit of blood transfused was associated with a 9% increase in the relative odds of HPgV acquisition (OR=1.09, 95% confidence interval, 1.06–1.11) [18]. Further, prevalence rates in cohorts of individuals with HIV infection range from 15% to 42% [3] (further discussed below). Additionally, the prevalence among patients who underwent transplantation for nonviral causes of liver disease was reported at 64% [54]. These findings are evidence of the increased risk factor for HPgV transmission in people in need of blood transfusions or exposed to blood and sexually transmitted infections. In general, HPgV is associated with a beneficial effect (i.e. less severe primary disease related to the coinfection) when paired with other infectious diseases with the most common being HIV. Recently, we also observed a possible salutary effect of HPgV coinfection also in the setting of COVID-19 infection [55]. Despite a substantial body of literature describing HPgV coinfections across diverse settings, no comprehensive synthesis has systematically evaluated the scope, consistency and clinical relevance of these findings. We therefore conducted a structured review of HPgV coinfection studies to characterize prevalence, genotype distribution across various geographic locations, population contexts, virological features, and reported clinical associations. By aggregating evidence across pathogens and study designs, we assess where effects are reproducible, context-dependent or absent, and propose a practical framework for interpreting the biological and clinical significance of HPgV in coinfections.

## Method

To identify all relevant studies on HPgV coinfection, defined as the concurrent presence of HPgV RNA and one other active infection, we conducted a comprehensive search of the PubMed database using the search string ‘(pegivirus OR HPgV OR HGV OR GBVC OR GBV-C OR ‘GB Virus C’ OR ‘GB Virus Type C’ OR ‘GB Virus-C’ OR HPgV OR HPgV-2 OR HPgV1 OR HPgV2 OR ‘human pegivirus’ OR ‘Hepatitis G’) AND (‘coinfection’ OR coinfection OR infection OR virus OR infections) AND (humans[MeSH Terms] OR human[tiab]) NOT (animals[MeSH Terms] NOT humans[MeSH Terms]) NOT review[Publication Type] NOT case reports[Publication Type].’ As of 03 February 2026, these searches yielded 1,561 results. From a total of 1,561 records, 138 were excluded due to a lack of full text, and 139 were excluded due to the lack of an English-language text. From there, 1,284 records were reviewed and excluded based on the following criteria: (1) no relation to HPgV-1, (2) no relevant or unclear coinfection prevalence data, (3) not a primary study, (4) case reports and (5) repeated cohorts. Records under the first exclusion criteria consisted of 73 studies relating to HPgV Type 2, non-human (i.e. chimpanzee) pegivirus, or other viruses. A total of 702 records were eliminated based on criterion 2 which comprised of studies with no active coinfection at the time of sampling, as patients were only screened for the presence of antibodies (*n*=215), mechanistic studies of the molecular and cellular interactions of HPgV with another virus (*n*=87), and studies in which no coinfection prevalence was reported or could be extracted (*n*=400). The third exclusion criteria were reviews, comments, editorials, or corrections of existing studies, comprising 149 search results. The fourth exclusion criterion eliminated 11 case reports with a sample size of 1. The final exclusion criterion eliminated three studies reporting secondary analyses of previously published cohorts that had already been included in this review to prevent duplication of participants.

After screening the search results, 346 independent studies were identified – Tables S1 and S2, available in the online Supplementary Material, provide detailed study level data. From there, each study was divided into the following categories: HCV, HIV, HBV, Torque Teno virus (TTV) and others (Fig. 1). We manually identified studies that reported drastically different results; those studies were further scrutinized for sources of discrepancies.

**Fig. 1.**
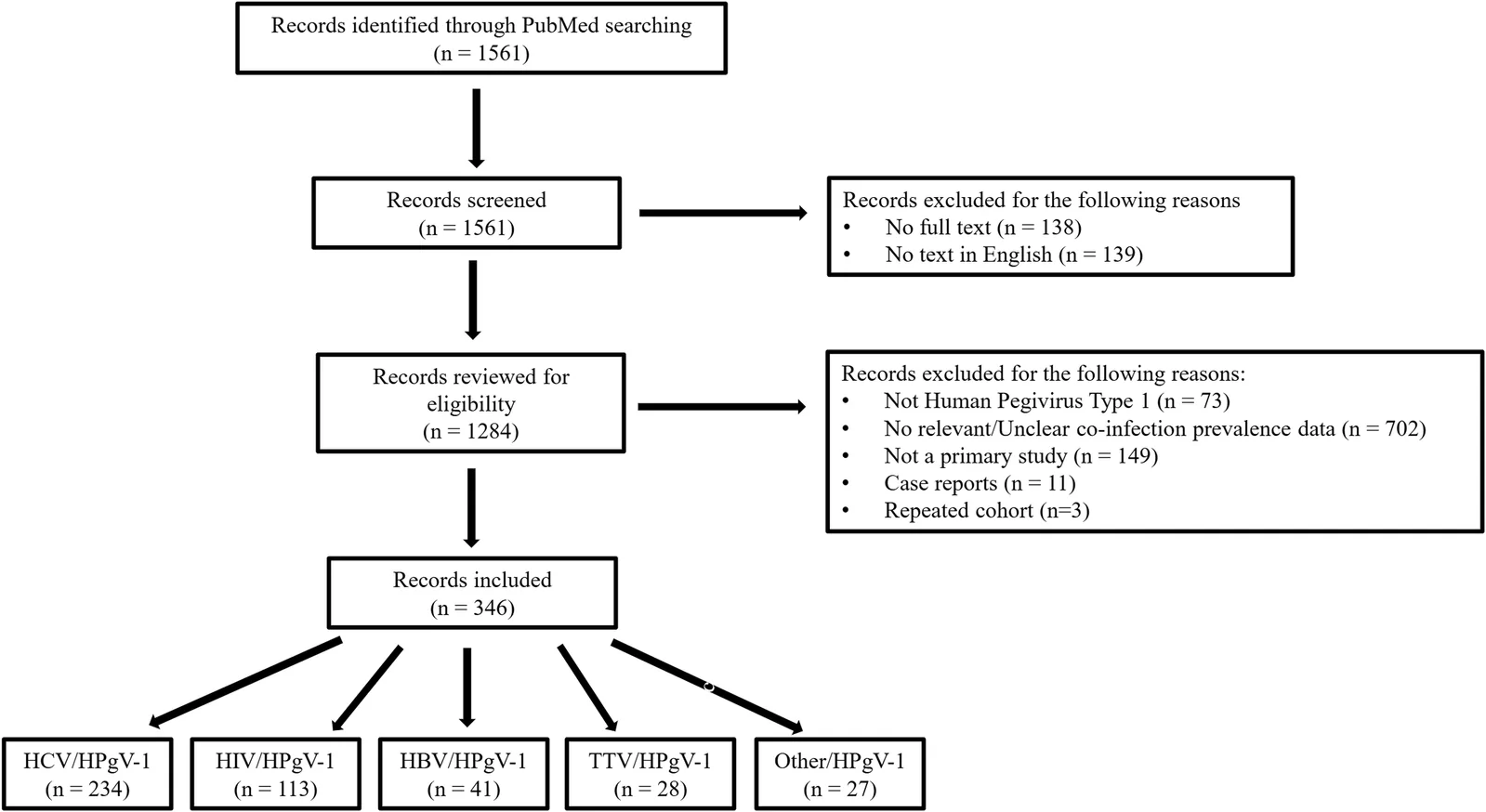
Overview of the Literature-mining approach. The number of articles retrieved at each step is noted in parentheses. Other HPgV-1 coinfections include EBOV, CMV, HEV, HDV, DV-2, HPV, HTLV-1, HAV and SENV. There are overlapping studies between the viruses. Hence, the total number of studies per coinfection type is greater than the number of relevant studies. EBOV*,* Ebola virus; CMV, cytomegalovirus; HEV, hepatitis E virus; HDV, hepatitis D virus; DV-2, Dengue virus serotype 2; HPV, human papillomavirus; HTLV-1, human T-lymphotropic virus type 1; HAV, hepatitis A virus; SENV, SEN virus.

This review provides a narrative synthesis of relevant literature to summarize and identify gaps in current knowledge. It does not follow a systematic review methodology, as it does not assemble and analyse studies with similar designs through predefined inclusion/exclusion criteria. For coinfections reported by multiple studies, the overall prevalence was calculated using a sample size-weighted average method by dividing the total number of positive cases across all studies by the combined sample size across all studies. This approach prevents the skewing of data by smaller sample sizes and gives greater weight to studies with larger sample sizes to provide the best descriptive estimate of the overall prevalence. Moving forward, the terminology ‘weighted average’ will be used when referencing the average prevalence of a coinfection.

### HPgV prevalence and genotype in coinfected cohort by geographic regions

Importantly, the magnitude of genetic divergence between HPgV genotypes is similar to mitochondrial DNA variation among human populations and does not imply biologically discrete or functionally stratified groups [3, 56]. Variations arise from single-nucleotide substitutions or deletions throughout the sequence [57], with nucleotide similarities ranging from 86.2 to 89.0% over the complete genome (reviewed in [58]). In this context, genotype-defining differences in HPgV are distributed throughout the 5′-UTR and translated genome regions, and recombination has been reported across multiple segments [57]. However, the biological consequences of these recombination events have not been systematically characterized, and there is currently very limited evidence [59] linking genotype or recombinant structure to distinct functional or clinical outcomes. These data support the interpretation of HPgV genotypes as phylogenetic groupings rather than functionally divergent viral forms.

Twenty four out of 346 studies included in this review reported HPgV genotypes in HIV, HCV and HBV coinfected cohorts (Fig. 1). Among those 24 studies, the overall HPgV coinfection prevalence with the aforementioned viruses ranges from 4.6 to 93.5% worldwide with genotype patterns varying substantially by region (Fig. 2); breakdown prevalence within each region described below is shown in Figure S1. It is important to note that differences in genotype sequencing methods, as well as the discovery of additional genotypes over time, may have contributed to variability in the genotypes reported across studies. In *Europe*, the overall coinfection prevalence is 23.1% while the predominant genotype is 2 (89.8%) [60, 61], determined from 118 HPgV-positive individuals across 2 studies. In *Asia*, the overall coinfection prevalence is 30.4%, with genotypes 7 (33.1%) and 3 (27.4%) as the most common [58, 62–67], determined from 474 HPgV-positive individuals across 7 studies. In *North America*, the overall coinfection rate is 19.4% with genotypes 1 (44.0%) and 2 (51.6%) as predominant [68], determined from 70 HPgV-positive individuals within 1 study. In *South America*, the coinfection prevalence is 22.6% with genotype 2 (72.2%) as the most common [69–73] determined from 191 HPgV-positive individuals across 5 studies. In *Africa*, the coinfection prevalence is 27.8% with genotypes 1 (44.6%) and 5 (37.7%) as the most common [74–78], determined from 130 HPgV-positive individuals across 5 studies. Finally, in the *Middle East*, the coinfection prevalence is 26.8% with genotype 2a (54.3%) as the predominant genotype [79–82], determined from 164 HPgV-positive individuals across 4 studies. While each geographic location has a different spread of genotypes identified, genotypes 2 and 2a appear to be the predominant subtype in most regions except Asia and Africa (Fig. 2). Since coinfection types are similar across all locations, non-HPgV infections are unlikely to explain the genotype discrepancy in Asia and Africa. Additionally, only three studies in healthy populations – conducted in Africa and Brazil – have reported HPgV genotype distributions, identifying genotype 1 and genotype 2 as predominant, respectively [83, 84]. These patterns are consistent with the genotype distributions observed in coinfected populations summarized in this review. However, whether these distributions are shaped by other infectious exposures or environmental factors remain unclear, owing to limited studies and scarce data from healthy populations.

**Fig. 2.**
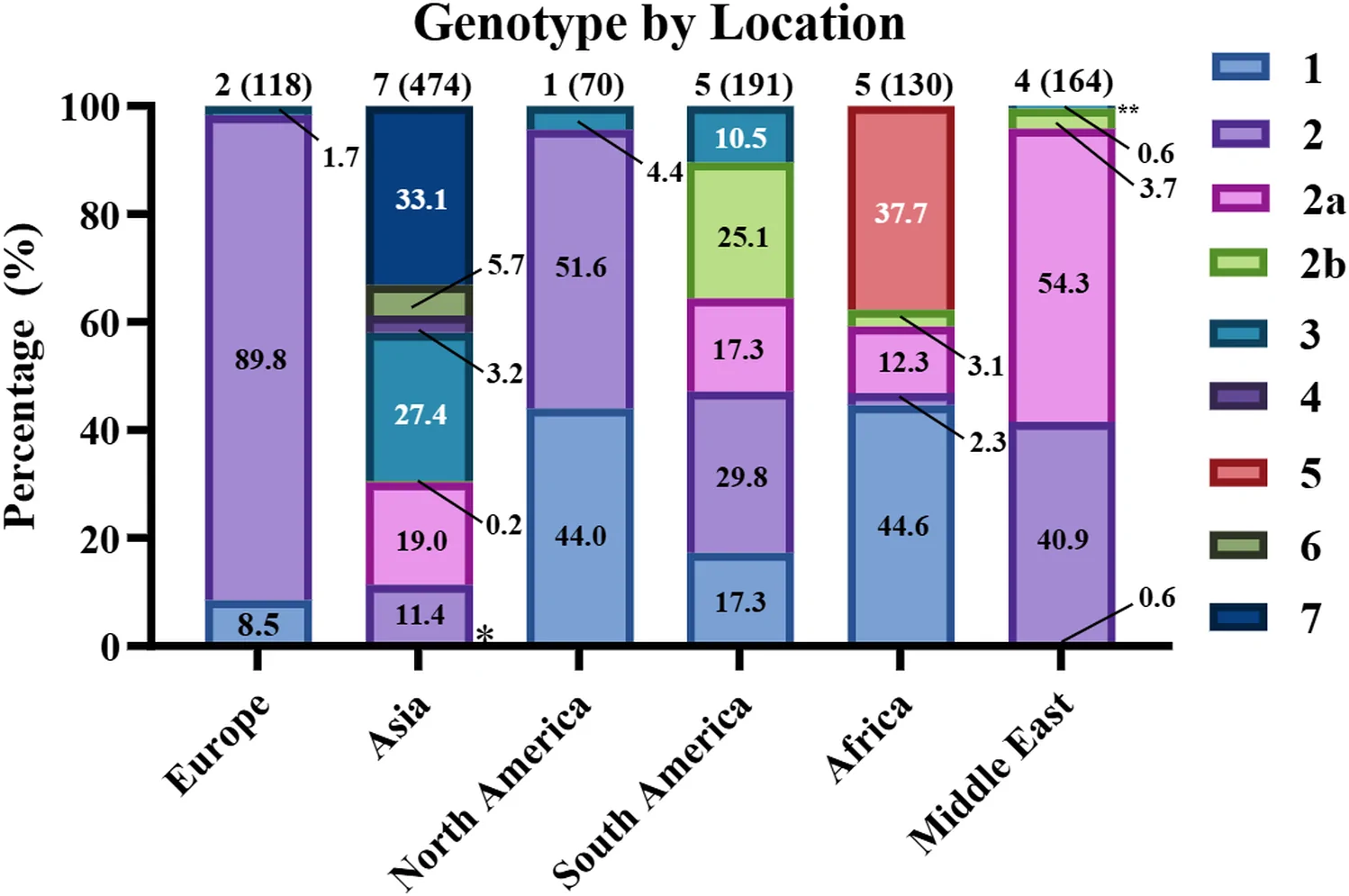
Breakdown of HPgV-1 genotype by geographic location. Note that some studies distinguished between genotypes 2a and 2b, while others grouped them together under genotype 2 since genotypes 2a and 2b were discovered after the publication of those studies. The percentage breakdown (%) is included within the bars, and the number of studies per location is indicated above each bar, with the total number of individuals in parentheses. *In Asia, genotype 2b had a prevalence of 0.2%. **In the Middle East, genotypes 1 and 3 each had a prevalence of 0.6% each.

Of the 24 studies reporting genotype in coinfected patients (Fig. 2), 3 studies highlight various factors linked to specific HPgV genotypes – namely, genotypes 2, 2a, 3 and 7 [63, 66, 85]. An Indonesian study noted that genotype 3 was associated with younger patients, while genotype 2a correlated more frequently with sexual contact [66]. In China, genotype 7 predominated among intravenous drug users, whereas genotype 3 was more common in heterosexual individuals [63]. Notably, patients with genotype 7 displayed lower HIV viral loads and higher CD4^+^ T-cell counts compared to HIV monoinfected controls, suggesting slower disease progression. A study conducted in the USA reported significantly higher IFNγy values for genotype 2 compared to genotype 1 from analysis of patient serums [85]. These mixed findings illustrate the complexity of genotype-specific effects, which likely depend on a combination of factors – transmission route, host immunity and regional epidemiology – that go beyond geography alone and are not consistently accounted for across studies, but thus far, no reproducible genotype-specific clinical phenotype has been established.

### HPgV and viral coinfections

#### HIV

Since the start of the acquired immunodeficiency syndrome (AIDS) epidemic over 40 years ago, HIV has been estimated to have infected 84 million people worldwide and is responsible for 40 million deaths. As a retrovirus, HIV is transmitted between humans via infected bodily fluids and enters CD4^+^ T cell lymphocytes through the CD4 receptor and other coreceptors, primarily C-C motif chemokine receptor 5 (CCR5) and CXCR4. Viral replication occurs in activated CD4^+^ T cells resulting in the progressive decline of CD4 cells and the development of AIDS if left untreated [86]. As detailed below, studies consistently show that HPgV RNA positivity (more so than anti-E2 antibody responses alone) is associated with higher CD4 counts, lower HIV viral loads and improved survival. These associations appear independent of antiretroviral therapy (ART) class but may vary by treatment era and clinical cofactors.

#### General prevalence and contributing factors

Since HPgV was first identified, 113 studies have examined HIV/HPgV coinfection. Among these, 101 studies reported HPgV viraemia measured at a single time point (i.e. cross-sectional), with a weighted average prevalence of 22.6% among HIV-infected individuals and a wide range (0–88.8%) (Fig. 3a). One study reported an exceptionally high prevalence of 88.8%, likely reflecting a cohort with extensive sharing of injection equipment [66]. Excluding this particular study reduces the weighted average prevalence to 22.3%, with 44 of 112 studies (39.6%) reporting prevalence between 21 and 30% (Fig. 3b). Further details regarding the studies reporting prevalence data can be found in Table S1.

**Fig. 3.**
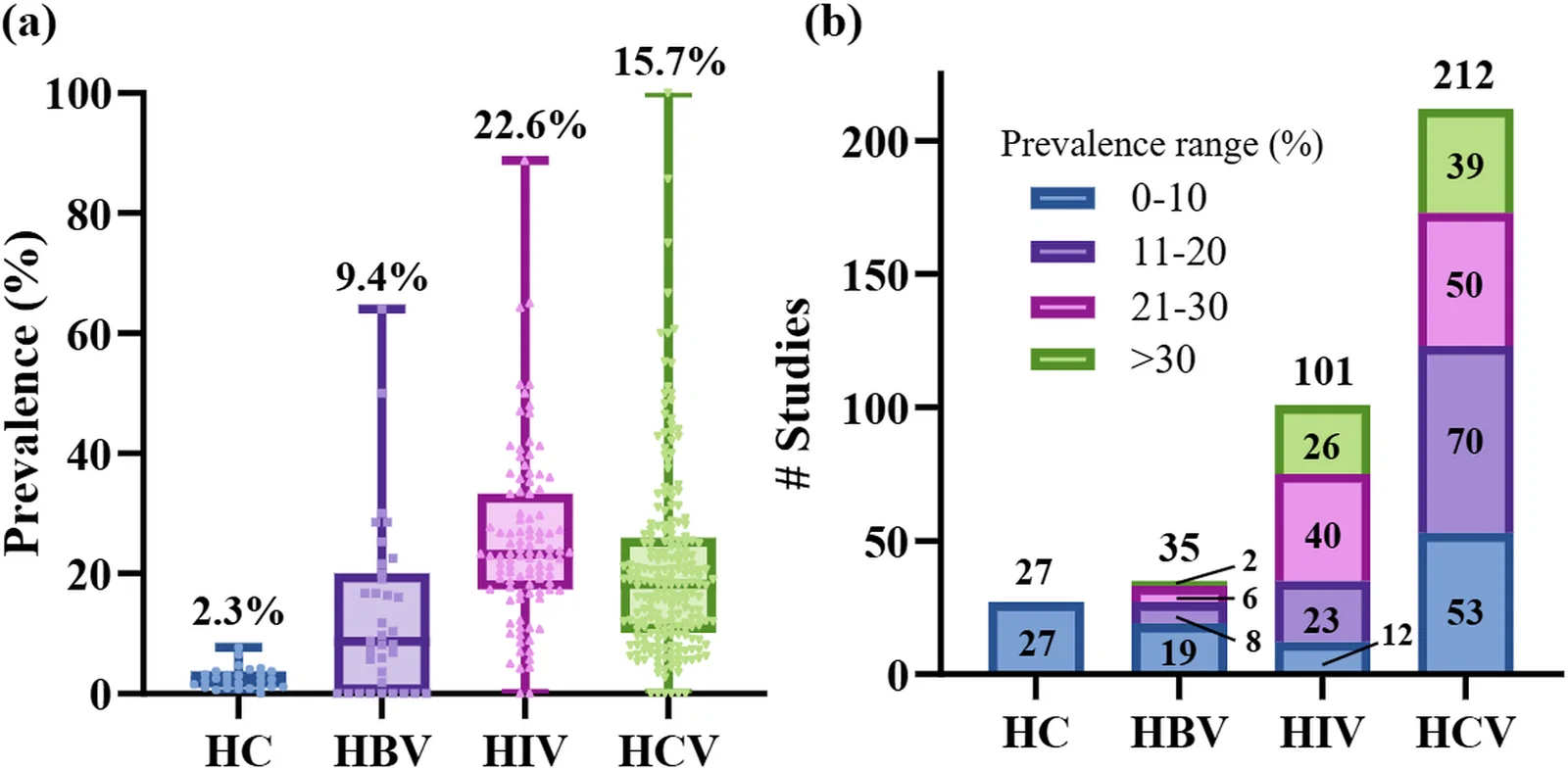
(**a**) Distribution of HPgV-1 coinfection prevalence. The box represents the IQR, and the whiskers represent the minimum and maximum data point for each group. The weighted average prevalence is noted above each bar. The mean HPgV-1 prevalence among healthy controls (HC) is 2.3% (median 2.1%, IQR [1.0%–3.4%]). Among those with HBV, HIV and HCV, the weighted average coinfection prevalence is 9.4% (median 8.7%, IQR [0.9%–19.5%]), 22.6% (median 23.1%, IQR [17.3–33.3%]) and 15.7% (median 18.2%, IQR [10.4–25.7%]), respectively. There are several clear outlying prevalence values for HBV/HPgV, HIV/HPgV and HCV/HPgV coinfection, although this is not surprising given the large number of studies across a wide variety of study designs. (**b**) Number of Studies per Coinfection Prevalence Range. All 27 studies reporting coinfection data for a healthy control population are within the 0–10% range. For HBV/HPgV coinfection, most studies report a prevalence rate below 10%. For HIV, there were 40 studies in which most reported prevalence were between 21 and 30%. For HCV, most studies were between 11 and 20%. *The total number of studies for HCV and HIV coinfection does not correspond to that in Fig. 1 because some studies reported HCV/HIV/HPgV multi-coinfection, and Fig. 3b only includes studies with HPgV/HCV or HPgV/HIV dual infection.

Notably, three of the five studies reporting the lowest coinfection rates (0, 5 and 5.7%) were conducted in paediatric cohorts with vertically acquired HIV, with median ages of 7.8, 2.2 and 10.9 years, respectively [87–89]. A study from Thailand reported that among 245 HIV/HPgV coinfected pregnant women, 101 infants (41%; 95% CI, 35–48%) while HPgV-positive, with maternal ART exposure was associated with increased vertical transmission. The same study found that HPgV transmission was significantly less frequent among HIV-infected infants than among HIV-uninfected infants (6.5 vs 46%) [90]. This reduced prevalence may reflect limited opportunities for HPgV acquisition through vertical transmission and/or interference by maternal HPgV anti-E2 antibodies, transferred transplacentally or via breast milk, which could impede the establishment of persistent infection. Together, these findings suggest that age, route of exposure, and immune maturation substantially influence observed HPgV prevalence. Longitudinal studies comparing HPgV viral load dynamics across childhood and adolescence would help clarify how HPgV persistence evolves as the immune system matures.

To avoid misinterpretation, it is important to emphasize that HPgV prevalence estimates in cross-sectional studies reflect viraemia measured at a single time point, not cumulative exposure or lifetime infection risk. Detection of HPgV RNA indicates ongoing viraemia, whereas individuals who were previously exposed but subsequently cleared infection would not be captured by RNA-based assays. Thus, the observation that approximately one-quarter of HIV-infected individuals are HPgV RNA positive does not imply limited exposure; rather, it reflects the balance between acquisition and spontaneous clearance.

Among the 113 studies reviewed, 60 investigated the relationship between age and HIV/HPgV coinfection, and 11 reported a significant association [17, 68, 69, 73, 88, 91–96], shown in Fig. 4a. Ten of these found that coinfected individuals, typically aged 29–39, were younger on average than those with HIV alone. The mean age for those individuals following this trend with HIV monoinfection and HIV/HPgV RNA coinfection were 37.1 and 33.2 [17, 69, 73, 91–95, 97], respectively. However, it is necessary to note that, although this difference is statistically significant, individuals aged 33–38 have very little biological differences. Other factors such as ART or number of sexual partners also did not seem to be correlated to the slight difference in age. In contrast, the singular study showing the opposite trend involved a perinatally HIV-infected paediatric cohort. Children in this cohort with HPgV coinfection were slightly older than those without (mean age 12.7 vs 10.7) [88]. The lower HIV/HPgV coinfection prevalence combined with the lower age for those with coinfection is not unusual, though, due to the significantly decreased lifestyle-related risk factors among children. For example, young children are less likely to engage in high-risk behaviours such as IDU or unprotected sex, which are common routes of HPgV transmission. Another notable finding was that patients with detectable HPgV anti-E2 antibodies were generally older than those with HIV monoinfection or HIV/HPgV RNA coinfection, aligning with the virus’s natural course of infection and seroconversion [17, 92, 93, 96]. However, antibodies are lost in many individuals post-seroconversion, so there is a potential underestimation of HPgV anti-E2 antibody prevalence in older individuals. Despite some evidence linking older age to HPgV viraemia in adults, the results are inconclusive; only 11 out of 60 studies found a significant association, indicating considerable variability in study populations, recruitment strategies, geographic regions and methodologies. The investigation of HPgV anti-E2 antibody prevalence in children also requires longitudinal follow-up or in-depth analysis between younger and older children due to the changing antibody response as the immune system matures with age. The wide range in study sample sizes (from 19 to 321 subjects) and the inconsistency in findings suggest that confounding factors, including risk behaviour and social determinants of health, may underline the observed trends. Therefore, age alone may not be a reliable predictor of HIV/HPgV coinfection, and future studies should account for behavioural and environmental exposures when assessing age-related patterns.

**Fig. 4.**
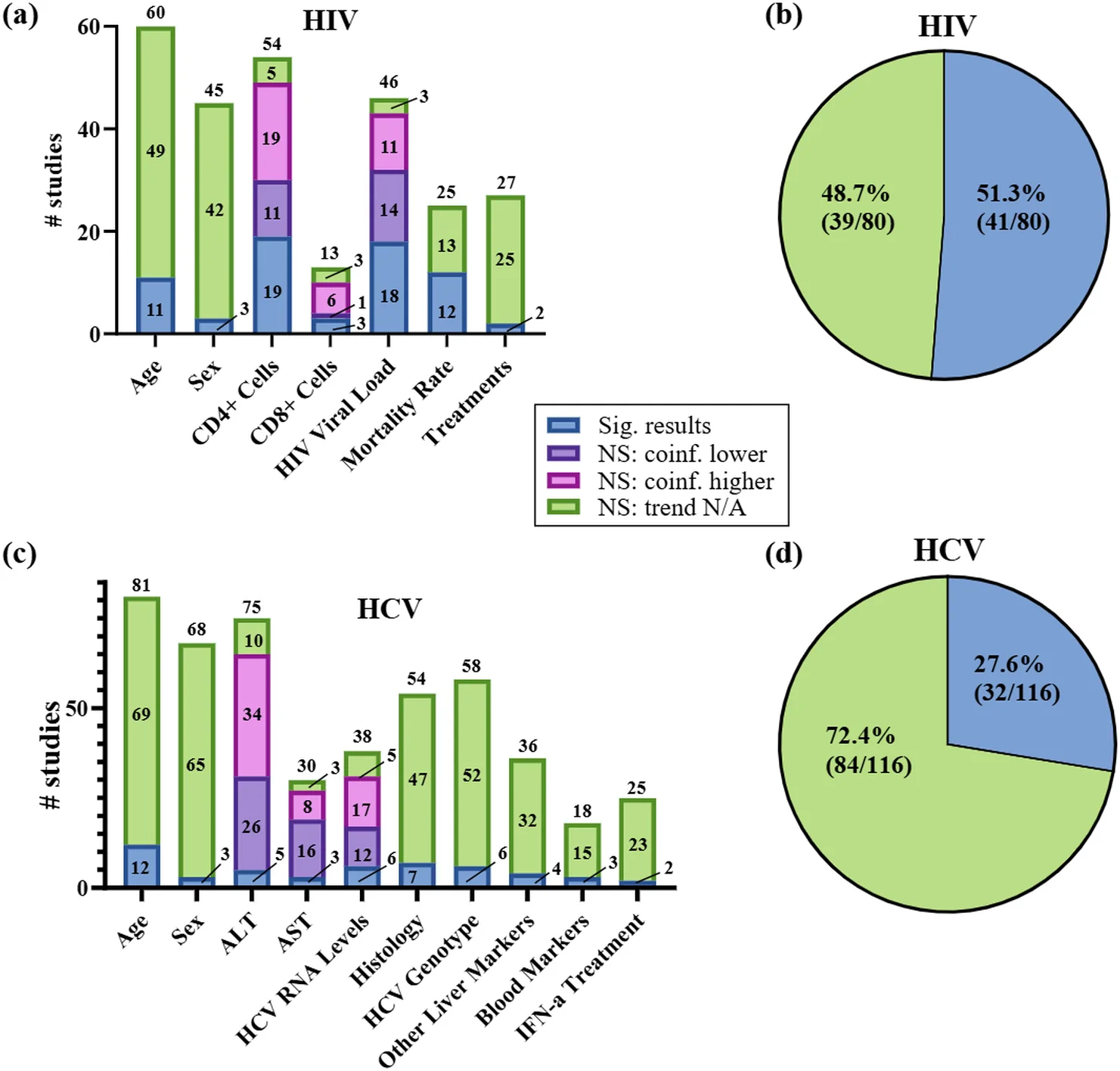
Summary of statistically significant and non-significant findings in studies of HIV/HPgV and HCV/HPgV coinfection. (**a**) Number of HIV/HPgV studies reporting statistically significant or non-significant findings for each factor examined. Non-significant findings are further categorized according to whether the value was lower or higher in the coinfected group than in the monoinfected group or whether no directional trend was reported. Mortality outcomes include mortality, progression to AIDS and progression to advanced CDC stages. Treatments include IFN, highly active antiretroviral therapy (HAART) and zidovudine. (**b**) Study-level summary of the 80 HIV/HPgV studies that compared at least one factor between HIV-monoinfected and HIV/HPgV-coinfected groups. Of these, 41 studies (51.3%) reported a statistically significant result for at least one factor shown in panel a, whereas 39 (48.7%) reported no statistically significant findings. (**c**) Number of HCV/HPgV studies reporting statistically significant or non-significant findings for each factor examined. The components of the histological findings, other liver markers and blood markers categories are detailed in Table S2. (**d**) Study-level summary of the 116 HCV/HPgV studies that compared at least one factor between HCV-monoinfected and HCV/HPgV-coinfected groups. Of these, 32 studies (27.6%) reported a statistically significant result for at least one factor shown in panel c, whereas 84 (72.4%) reported no statistically significant findings. Because individual studies could evaluate multiple factors, they may contribute to more than one category in panels a and c. The study totals in panels b and d differ from those in Figs 1 and 3b because these panels include only studies that directly compared monoinfected and coinfected groups. All panels share the same colour legend.

Among the 113 studies reviewed, 45 examined the relationship between subject sex and HPgV coinfection rates, only 3 found a significant difference [17, 94, 95], as seen in Fig. 4a. Two studies reported a higher percentage of males with HIV/HPgV coinfection, while one study reported the opposite trend. Two of the three studies also saw a significantly higher number of patients with a history of IDU and identifying as homosexual or as men who have sex with men (MSM), both of which are common risk factors for the contraction of HPgV and other viral processes [17, 94]. However, many HIV/HPgV coinfection studies are centred around a male cohort where common risk factors include IDU and MSM. Thus, the disparity in these findings could be influenced by the male-biassed enrollment in HIV clinical trials, reflecting the higher prevalence of HIV among males. This larger male sample size may skew the results, making it challenging to draw definitive conclusions about the role of gender in HPgV coinfection. Therefore, while some evidence suggests sex-related differences, the overall data are inconclusive and may be influenced by study design and demographic factors.

#### HIV virology and pathology

HIV virology and pathology appear to be notably influenced by HPgV coinfection, with effects on CD4^+^ T-cell counts, HIV RNA viral load and patient mortality. Out of 113 studies, 32 reported beneficial outcomes of HPgV/HIV coinfection, including prolonged survival [17, 19, 62, 63, 70, 73, 76, 81, 88, 93–96, 98–116]. Fig. 4a describes the number of studies reporting significant versus non-significant findings for commonly studied variables, while Fig. 4b displays the overall distribution of studies comparing monoinfection and coinfection that report significant associations with HIV/HPgV coinfection. Additional factors, such as CCR5 expression on CD4^+^ and CD8^+^ T cells, were also reported to be significantly associated with HIV/HPgV coinfection. However, these variables are not shown in Fig. 4a since there are a limited number of studies examining these markers and are not routinely evaluated among HIV cohorts. More details can be found in Table S2. Of the 113 studies reviewed, 32 reported statistically significant beneficial associations – particularly with CD4^+^/CD8^+^ cell counts, HIV viral load and clinical outcomes – while an additional 30 studies demonstrated concordant trends that did not reach statistical significance. Taken together, the consistency and direction of these findings reinforce HPgV’s lymphotropic properties and provide a compelling rationale for investigating gene therapies or vaccines that harness its biological interactions with HIV.

#### CD4^+^ and CD8^+^ T-cell counts

Among 54 studies focusing on CD4^+^ T-cell counts in HIV/HPgV coinfection, 19 detected a significant difference compared to HIV monoinfection [17, 19, 63, 70, 76, 81, 93, 96, 98–102, 104–107, 114, 116]. Of those, all but two studies observed higher CD4^+^ counts in coinfected patients. Of the 35 studies that did not find a statistically significant difference, 51.4% of them (18/35) consisted of CD4^+^ trends in the same direction as the 17 studies mentioned above. Because elevated CD4^+^ counts indicate a more robust immune response and are commonly used to monitor HIV progression and ART effectiveness, despite 36 studies showing no significant impact, the cumulative observations still suggest that HPgV coinfection may slow disease progression and contribute to the improved survival observed in many studies. Of note, two longitudinal studies reported a higher CD4^+^ T-cell counts over the entire course of 11 months [100] and 17 months [96] when compared to the initial visit, suggesting that HPgV may confer sustained immunological benefits beyond early exposure. However, no studies have measured CD4^+^ or CD8^+^ T-cell counts before and after documented acquisition of HPgV, limiting causal inference. Moreover, the absence of continued CD4^+^ increases between consecutive visits suggests a plateau effect, indicating a potential upper limit to HPgV’s capacity to augment CD4^+^ T-cell counts. Four studies also examined CD4+ cell counts among patients with HPgV anti-E2 antibody [17, 93, 96, 99]. Two of them found that those with persistently positive HPgV RNA had higher CD4^+^ cell counts than those who were only HPgV anti-E2 antibody positive, suggesting greater benefits when active viral infection is present [17, 96]. Additionally, one study found a lower expression of Ki67 on CD4^+^ T cells for those with HIV/HPgV coinfection, in conjunction with the higher CD4^+^ cell count [105]. The combination of these factors with a higher CD4^+^ cell count further suggests that HIV coinfection with HPgV works to suppress HIV infection.

Importantly, one of the observations of lower CD4 cell counts derives from a cohort receiving IL-2 treatment, introducing additional biological context that complicates interpretation of this finding relative to studies conducted without IL-2 exposure [114]. This 2009 study found that HPgV negatively interferes with CD4 expansion in response to IL-2 therapy, indicating that HPgV status should be considered when determining treatment plans for HIV. On the other hand, another study in 2003 reported fewer ART failures among those with HIV/HPgV coinfection [100, 101]. These studies are complicated as their cohorts are comprised of individuals with different transmission risks, duration of infection and varying follow-up. Consequently, these results underscore the need for further research into harnessing HPgV’s beneficial properties to inform or enhance HIV treatment strategies.

Among 13 studies investigating CD8^+^ T-cell counts, 3 reported significantly higher counts in coinfected individuals [88, 93, 100]. Even though the other 10 studies did not reach significance, 85.7% (6/7) of the 7 studies with quantifiable data followed the trend of a higher CD8^+^ count for those with coinfection. Observations of higher CD4^+^ and CD8^+^ T-cell counts in HPgV-positive individuals strengthen the view that HPgV coinfection can confer immunological benefits to people living with HIV. Many studies show favourable trends that do not reach statistical significance – limitations largely attributable to small cohorts and heterogeneous study designs – yet the collective pattern points to a multifactorial mechanism in which HPgV interacts with host and viral factors to help restrain HIV pathogenesis.

#### HIV RNA load

Among 46 studies examining the relationship between HIV RNA viral load and HPgV coinfection, 18 reported a significant correlation. In 16 of these, coinfection was linked to lower HIV viral loads [17, 63, 70, 73, 81, 93, 95, 96, 100–103, 106, 107, 112, 115], indicating that HPgV may enhance immune function and suppress active HIV. Additionally, one of the 16 studies reported a statistically significant inverse correlation (R=−0.57) between HPgV viral load and HIV viral load at a singular time point, but significance was lost when analysing the longitudinal data due to the lower effect size and loss of many continuous HIV/HPgV viral load pairs [81].

The other two studies producing contradictory findings reported a higher HIV viral load upon HIV diagnosis in those with HPgV coinfection [108, 113]. In one of these, all participants were newly diagnosed with both HIV and HPgV [113], suggesting that HPgV may need sustained infection before offering protective benefits. The other outlying study consisted of an HIV cohort who just began ART, indicating differences in HPgV interaction with HIV at the start of a new treatment [108]. For the other 28 studies with no significant results, 14 and 11 studies reported trends of lower and higher HIV viral loads, respectively, for those with HIV/HPgV coinfection. Although most of these studies consisted of patients with chronic HIV infection, the exact duration of infection is unclear for many which could explain the differing relationships between HIV viral load and HPgV status.

Additionally, one longitudinal study spanning 5 years reported that HIV viral loads in coinfected patients remained consistently lower than in monoinfected individuals [107]. Finally, in three investigations that distinguished between HPgV anti-E2 antibody positive and actively infected patients, those with active HPgV infection consistently showed lower HIV viral loads, reinforcing the notion that ongoing HPgV viraemia confers more pronounced benefits [17, 93, 96]. Collectively, these findings suggest that HPgV can dampen HIV replication throughout the course of infection; however, the strength of this benefit seems to depend on factors such as the infection stage and coinfections like HCV, underscoring the need for further investigation.

#### Mortality rate and HIV treatments

Among 25 studies looking at the effects of HPgV on mortality rate and the progression towards AIDS, 12 out of 25 (48.0%) studies found a significant difference when comparing HIV monoinfected and HIV/HPgV coinfected individuals [17, 19, 76, 81, 93, 96, 101, 106, 107, 109–111]. In all 12 studies, coinfected individuals demonstrated a notably lower mortality rate or progression towards AIDS than those with HIV alone. Consistent with observed trends in viral load and CD4^+^ T-cell counts, actively coinfected patients also experienced a greater survival benefit compared to those who were only HPgV anti-E2 antibody positive [17, 93]. Furthermore, three of these studies reported significant findings even after adjusting for values such as baseline CD4 count, ART status and HIV viral load [19, 93, 96, 110], providing stronger evidence towards the beneficial effects of HPgV coinfection with HIV mortality. Among 27 studies that investigated HIV treatments, only 2 studies found significant differences between HIV monoinfected and HIV/HPgV coinfected individuals. Additionally, one study found greater success in response to highly active antiretroviral therapy (HAART) among patients with HIV/HPgV coinfection [100], while another study reported fewer patients with HAART exposure among those with HIV/HPgV coinfection [96]. Comparisons of HIV treatment outcomes and associations with HPgV infected are particularly nuanced, as differences in immune status, treatment plans and HIV disease progressions can vary greatly between patients, further complicating comparisons.

#### Mechanisms of HPgV interference on HIV infectivity

Although the cellular crosstalk between HPgV and HIV is not yet fully charted, the evidence gathered to date already provides a solid springboard for envisioning – and starting to design – novel therapeutic strategies. Early studies demonstrated that HPgV incubation with primary CD4 cells decreased HIV-1 co-receptors CCR5 and altered expression of chemokines that inhibit HIV entry [94, 105, 117, 118]. In addition, HIV replication requires T cell activation and proliferation to proceed, and the strength of the TCR signal is proportional to the level of HIV-1 replication [119]. HPgV infection blunts TCR-mediated signalling and T-cell activation *in vivo* [105, 120] and *in vitro* [121]. HPgV’s E2 protein contains a highly conserved peptide motif containing a predicted tyrosine kinase substrate site sufficient to inhibit TCR-mediated activation of tyrosine kinase [121]. By reducing TCR signaling and immune activation, the effects of HPgV on CD4^+^ T cells contribute to reduced HIV-1 replication. Little is known regarding potential direct viral effects of HPgV on HIV-1 replication; however, current research centres on HPgV E2’s interaction with HIV-1 gag, but E2 itself also exerts direct effects by inhibiting HIV-1 assembly and release (particularly CAp24 and MAp17) through the downregulation of ADP ribosylation factor 1 – an essential factor for Gag membrane localization and particle production [122, 123]. It is important to note that any direct interactions between HPgV and HIV-1 would require HPgV-infected cells to release viral products taken up by HIV-1-infected cells, since the number of HPgV genomes is low (0.001–1 genome equivalent per CD4^+^ T cell in people living with HIV-1 with HPgV coinfection [124]) and only ~1 in 10,000 CD4^+^ T cells contains replication-competent HIV-1 proviral DNA genomes [125]. Additionally, the N-terminal region of HPgV E2 shares sequence similarities with HIV-1 gp120, facilitating an interaction with the gp41 disulfide loop that inhibits viral fusion [126]. These molecular parallels and protein–protein interactions could pave the way for next-generation HIV entry inhibitors that leverage HPgV’s unique antiviral mechanisms. Recent studies provide further support for HPgV’s therapeutic potential. For instance, Herrera *et al*. demonstrated that the HPgV E2 protein sequence interacts with HIV-1’s target fusion peptide, modifying its conformation [127]. Taken together, these findings underscore HPgV’s capacity to reduce HIV pathogenesis and suggest a novel avenue for developing vaccines or adjunctive treatments to lower HIV/AIDS morbidity and mortality. However, the lack of an *in vitro* human replication system of HPgV poses as a barrier to directly study interactions between HPgV anti-E2 antibody with HIV. These insights naturally prompt the question of which other viral pathogens – or immune-driven diseases – might likewise be tempered by HPgV’s immunomodulatory arsenal.

### HCV

HCV has the largest number of HPgV coinfection reports to date, with 234 published studies, 10 of which reported significant associations with factors such as liver function markers [aspartate aminotransferase [AST] and alanine aminotransferase (ALT)] and HCV RNA levels [36, 72, 128–135]. Given that HPgV was initially thought to be part of the hepatitis virus family, these studies sought to clarify its relationship with other common hepatitis viruses. Across the 234 studies, 212 reported data on the prevalence of active HPgV infection among HCV-infected patients, while the remaining studies focused on other factors such as multi-coinfection. The weighted average prevalence of active HCV/HPgV coinfection at a single time point across the 212 studies was 16.9%, ranging from 0 to 100% (Fig. 3a). At the study level, most reports clustered at lower prevalence ranges, with 53 of 212 studies (25.0%) reporting coinfection rates of 0–10% and 70 of 212 studies (33.0%) reporting rates of 10–20% (Fig. 3b). Further details can be found in Table S1. Three studies reported particularly high or low prevalence rates, but no clear factors were identified to fully explain these outliers. Despite these extremes, no consistent, overarching explanation emerged for the wide variability, underscoring the complexity of HCV/HPgV coinfection and the need for further investigation. Importantly, these cross-sectional estimates reflect point prevalence and do not inform the likelihood of HPgV acquisition or persistence following exposure. Fig. 4c describes the number of studies reporting significant versus non-significant findings for commonly studied variables, while Fig. 4d displays the overall breakdown of studies comparing monoinfection and coinfection that report significant findings associated with HCV/HPgV coinfection. Additional variables, such as platelet count and mean corpuscular haemoglobin, were also reported to be significantly associated with HCV/HPgV coinfection. However, they are not displayed in Fig. 4c due to the limited number of studies examining these markers since they are not routinely examined in HCV patients. More details regarding these markers can be found in Table S2.

### Age and sex differences

Of the 81 studies examining age, only 12 found a statistically significant distinction between HCV monoinfected and HCV/HPgV coinfected individuals. In 10 of these, coinfected patients were significantly younger than those with HCV alone, typically aged 32–53. The mean age for those individuals following this trend with HCV monoinfection and HCV/HPgV RNA coinfection were 47.6 and 39.1 [132, 136–144]. No specific factors were identified to explain the discrepancy of the other two studies [145, 146]. Regarding sex, only 3 of 68 studies addressing this variable reported a significant difference [35, 147, 148], reporting conflicting results. Thus, the limited number of positive reports precludes firm conclusions regarding sex predilection. Importantly, these analyses are largely cross-sectional and heterogeneous in design, limiting the ability to exclude confounding by unmeasured demographic or clinical factors.

### Clinical biomarkers of liver disease

Among the 234 studies examining HCV/HPgV coinfection, relatively few reported significant differences in liver injury markers: 5 of 75 studies assessing ALT [128, 129, 132–134] levels and 3 of 30 assessing AST levels [132, 133, 135]. If HPgV conferred a protective effect analogous to that observed in HIV coinfection, one might expect consistently lower ALT and AST levels among HCV/HPgV coinfected individuals. However, ALT and AST primarily reflect hepatocellular injury and inflammation and do not reliably correlate with HCV viral replication or immune control, unlike CD4^+^/CD8^+^ T-cell counts in HIV. Consistent with this biology, findings across studies were heterogeneous and directionally inconsistent. Among studies reporting significant associations, four out of five reported higher ALT levels in coinfected patients [128, 129, 133, 134] and two of the three reported lower ALT levels [132, 133]. Patterns among non-significant studies with quantifiable data were likewise mixed: 56.7% (34/60) reported higher ALT levels in coinfected patients, while 66.7% (16/24) showed trends towards lower AST. Taken together, the absence of consistent directionality or magnitude across studies does not support a meaningful effect of HPgV on ALT or AST levels in HCV infection.

As for HCV RNA levels, 6 of 38 studies that evaluated HCV viral load found significant differences between monoinfected and coinfected individuals. Three out of six reported a lower HCV viral load in coinfected patients [72, 129, 131], while the other half identified a higher viral load [36, 128, 130]. Among the non-significant studies with quantifiable data, 56.0% (14/25) of them trended towards higher viral loads for individuals with coinfection. Overall, the variable natural history of HCV infection, the lack of predictive serum and virologic measurements and contradictory results for AST, ALT and HCV RNA do not suggest a consistent effect of HPgV infection on HCV infection outcomes. Given that most studies did not identify significant changes, it is reasonable to conclude that hepatic release of AST and ALT is not consistently associated with HPgV coinfection status.

### Histological findings

Because HCV infection is closely linked with liver disease, many studies focus on histological indicators – such as bile duct damage and fibrosis – to assess liver function. Among 55 investigations that reported histology in cases of HCV/HPgV coinfection, 7 found significant differences in histology compared to HCV monoinfection [137, 142, 143, 146, 149–151]. These findings suggested heterogeneous effects of HPgV on liver outcomes. While some studies reported greater bile duct damage, higher degrees of inflammation and greater irregular generation [137, 142, 146, 149, 150], other studies reported lower rates of graft hepatitis cases and advanced fibrosis [143, 151]. However, these studies are limited by the unknown durations of HCV infection – an important confounder, as longer infection duration is associated with greater histologic severity and may also increase cumulative opportunity for HPgV exposure. The low number of significant findings combined with the lack in trend of blood markers such as AST and ALT levels do not point to a clear direction regarding HCV pathogenesis. Thus, further research into their connection with HPgV is needed to enhance our understanding of both lesion development and the mechanisms by which HPgV influences immune responses in the context of liver damage.

### HCV genotype

HCV is classified into at least eight genotypes (numbered 1–8) that might potentially influence disease progression, treatment strategies and clinical outcomes [152]. Among the 58 studies evaluating HCV genotype among those with HCV/HPgV coinfection, only 6 reported significant differences between HCV genotype and HPgV coinfection compared to HCV monoinfection [128, 136, 138, 143, 147, 153] (of note, none of these studies sequenced HPgV to determine the genotype). These six studies indicated that HCV genotypes 1b, 2b and 3a were more common in coinfected individuals. These findings differ from the most prevalent HCV genotype of their respective countries in five of six of these studies [154], indicating a more complex relationship at play. However, the small number of studies reporting any significant findings and the inconsistencies in the studies that report significant differences makes it likely that there is little association between HCV genotype and HPgV.

### HBV

HPgV prevalence among HBV-infected patients has been examined across 35 independent studies, yielding a weighted average of 9.4% (range: 0–45.2%) (Fig. 3A) with 54.3% of studies falling within the 0–10% range (Fig. 3B). The two studies with the highest prevalence rates of 50.0 and 64.0% could be due to potential improper screening of blood bank, as discussed in the original study, and a high number of transfusions, respectively [40, 155]. However, excluding these studies did not substantially change the overall prevalence mean (8.2%). Beyond these prevalence figures, there were no studies reporting significant findings, although several studies investigated AST and ALT values with no significant findings. Thus, HPgV’s true impact on HBV-related liver damage remains uncertain.

### TTV

TTV is a DNA virus identified as a possible causative agent of post-transfusion hepatic injury [156]. Twenty-six studies have investigated the prevalence of HPgV in TTV-positive individuals, reporting a weighted average prevalence of 10.9%, similar to that of HBV. The data ranges from 0 to 45.2% with most (73.1%) studies reporting rates below 20.0%. However, none of these studies reported any statistically significant associations with clinical variables with coinfection. Taken together, the limited number of studies, wide variability in prevalence estimates and near absence of clinical correlations preclude meaningful conclusions regarding TTV/HPgV coinfection. Further investigation would be required to determine whether any biological or clinical interaction exists between these viruses.

### EBOV

Across two studies, the weighted average prevalence of HPgV among individuals infected with EBOV (Ebola virus) averaged 19.3% [157, 158]; however, it is important to note that no additional viral testing was performed, including HIV, HCV and HBV. Among EBOV-coinfected patients, the survival rate was 53% compared to the 22% rate of those without HPgV [157]. Correlation was observed between age, survival and HPgV status: younger patients (ages 21–45) had higher rates of HPgV infection and better survival outcomes, while older patients had lower survival rates. However, in a multivariate analysis considering age, mortality and HPgV status together, the significance of HPgV status diminished, suggesting age as a confounding variable. Mechanistically, although EBOV and HPgV infect distinct immune cell populations and do not directly interact, their effects on host immune regulation may converge in biologically meaningful ways. HPgV has been associated with reduced T cell activation and lower proinflammatory cytokine levels in HIV infection. Given that EBOV pathogenesis is characterized by extensive T cell loss and dysregulated cytokine production, similar immune-modulatory effects of HPgV could plausibly mitigate immunopathology and contribute to improved survival among EBOV patients coinfected with HPgV.

### Other infections

Additionally, 11 studies investigated HPgV coinfection prevalence with a variety of other viruses including cytomegalovirus, hepatitis E virus (HEV), hepatitis D virus (HDV), Dengue virus serotype 2 (DV-2), human papillomavirus, active Human T-Lymphotropic Virus-1, hepatitis A virus and SEN virus. These 11 studies reported a coinfection prevalence of 37.3, 33.3, 29.6 (two studies), 25.0, 16.7, 5.4, 2.4 and 1.5% (two studies), respectively [46, 49, 159–167]. Interestingly, one other study to note is a 2021 controlled human malaria infection (CHMI) study conducted in Tanzania and Equatorial Guinea that reported greater protective effects of the malaria vaccine for those with HPgV compared to those without HPgV (62.5% vs 51.6%) [168]. This overall protection was evaluated by the presence or absence of asexual blood-stage parasitaemia following CHMI. However, the malaria/HPgV coinfection prevalence could not be determined, since the data included multiple sampling time points before and after CHMI without specifying which time points yielded positive results. Therefore, this study is not included in the total final count of 346 relevant HPgV coinfection studies. Although malaria is caused by a protozoan parasite rather than a virus, it provides a relevant context to examine HPgV coinfection, as malaria profoundly alters host immune regulation. Including non-viral infections is therefore essential for a comprehensive assessment of HPgV’s immunomodulatory effects across diverse infectious settings. For many of these viruses, prevalence estimates are currently based on a single study and, therefore, may not yet be broadly generalized.

### Multi-coinfection

Although multi-coinfections involving HPgV, HIV, HCV, HBV and other viruses are noted in Fig. 5, most entries reflect data from only a single study, limiting our ability to draw reliable prevalence estimates. From the limited available research, coinfections involving HCV or HBV alongside HIV/HPgV appear to share similar prevalence rates to HIV/HPgV alone, suggesting no strong additive effect. The additional infection of either HCV or HBV does not seem to drastically change the prevalence of HIV/HPgV coinfection. When HIV is added to HCV/HPgV or HBV/HPgV coinfection, prevalence may rise to levels comparable to HIV/HPgV, potentially due to HIV-induced immunodeficiency that facilitates HPgV persistence. IDU, involving shared needles and other bodily fluid exposures, also emerges as a significant risk factor. As HPgV can persist over long periods, those with a history of any additional viral infections may show increased chances of multi-coinfection. Interestingly, one study reported that individuals with HIV/HCV/HPgV triple-infection demonstrated significantly more cirrhosis and hepatic encephalopathy [169], while another study reported less Scheuer stage 3 inflammation and Scheuer stage 4 fibrosis for individuals with triple infection [170]. Ultimately, more studies are needed to clarify how HPgV behaves in the context of multiple concurrent infections.

**Fig. 5.**
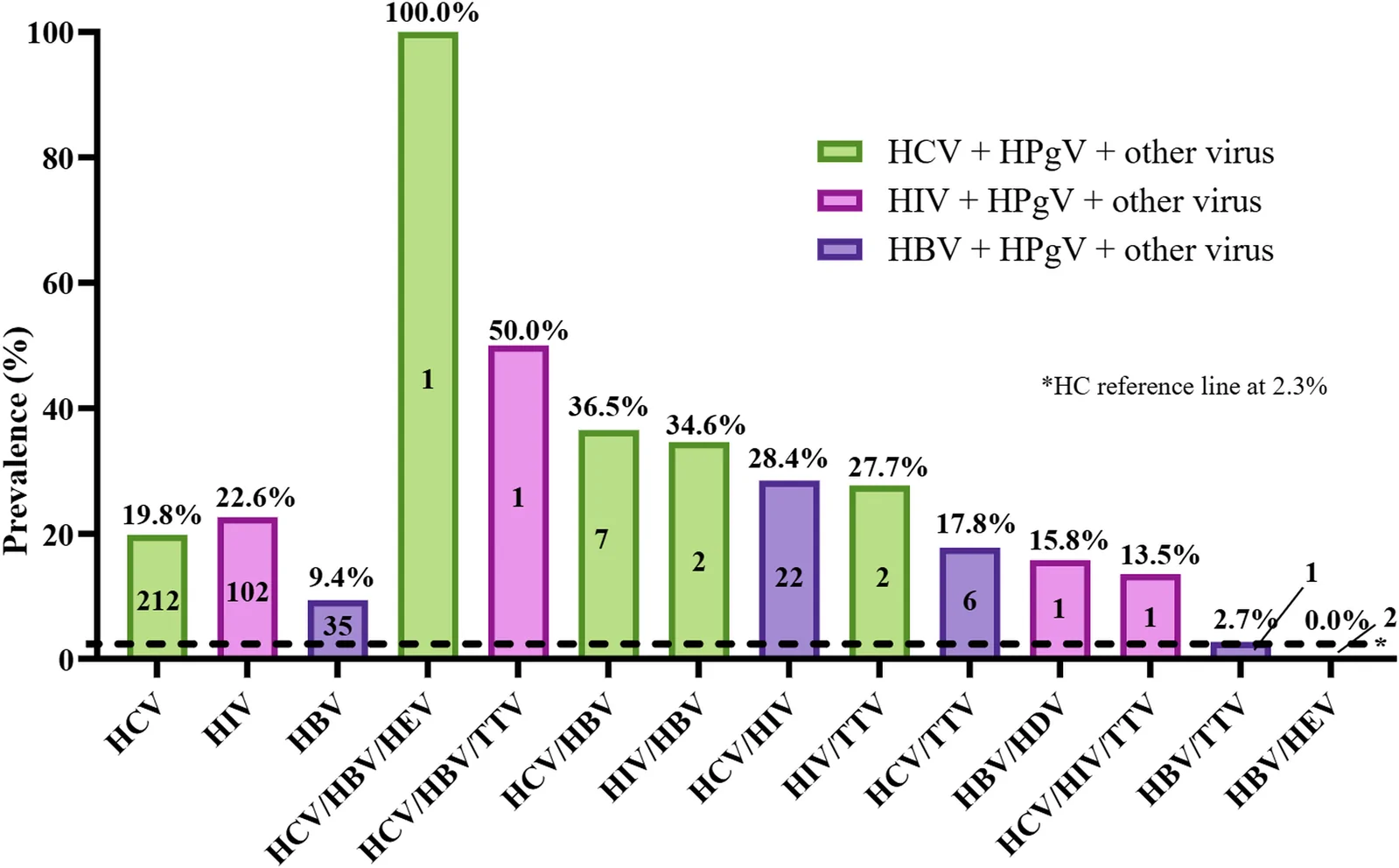
Prevalence of different multi-coinfections with HPgV-1. The number of studies per multi-coinfection is indicated within each bar, and the weighted average prevalence rate is indicated above each bar. Additionally, the total number of patients investigated across all studies per infection are as follows: HCV (*n*=17,329), HIV (*n*=22,494), HBV (*n*=1,962), HCV/HBV/HEV (*n*=1), HCV/HBV/TTV (*n*=2), HCV/HBV (*n*=52), HCV/HIV (*n*=2025), HIV/TTV (*n*=65), HCV/TTV (*n*=152), HBV/HDV (*n*=19), HCV/HIV/TTV (*n*=37), HBV/TTV (*n*=37) and HBV/HEV (*n*=2).

### Conclusion

This review consolidates the current evidence on HPgV coinfection, which presents a complex and often contradictory picture. While some consistent trends point to a protective, immunomodulatory role, particularly in HIV, most studies do not reach statistical significance, potentially due to wide variability in study design and confounding factors. Nonetheless, consistent trends across subsets of studies support a role for HPgV in modulating host immune responses and influencing HIV or HCV disease trajectories. Its selective association with certain NHL subtypes further suggests a context-dependent immunomodulatory or lymphotropic role, which may be protective in viral coinfections but potentially pro-oncogenic in dysregulated immune environments. This bivalent behaviour remains poorly understood and would benefit from mechanistic studies using newly emerging *in vitro* models and functional assays. Temporal ambiguity and residual confounding – particularly related to transfusion exposure and immunosuppression – limit causal inference across many HPgV coinfection studies. Future prospective sero-incidence studies or genetically informed approaches, such as Mendelian randomization, may help address these gaps. Given the high global prevalence of HPgV, its detection in diverse clinical populations, including patients with unrelated diseases, is expected and inevitably yields numerous statistical associations. In several instances, such associations have been extended towards mechanistic interpretations despite limited control for confounding, small sample sizes or insufficient virologic quantification. Accordingly, HPgV detection alone should be interpreted with caution, and pathogenic roles should not be inferred without rigorous epidemiologic and mechanistic validation.

This review underscores several methodological gaps, including wide variability in study designs, inconsistent reporting of HPgV genotypes, lack of standardized clinical endpoints and limited longitudinal follow-up. The lack of an *in vitro* replication system for HPgV and the lack of an animal model coinfection system with similar retroviral outcomes has hampered the field. Genotype-specific effects remain underexplored and are often confounded by geographic and behavioural factors. Future studies should prioritize harmonized protocols, larger and more diverse cohorts and stratification by genotype, transmission route and immune status to clarify HPgV’s true biological impact. Addressing these gaps is essential to determine whether HPgV is a benign bystander, an immunomodulatory ally or a context-dependent pathogen and whether its properties can be harnessed for therapeutic or preventive interventions in HIV, HCV and other viral infections.

## Supplementary material

10.1099/jgv.0.002365 Fig. S1.

10.1099/jgv.0.002365 Table S1.

## Abbreviations

AIDS: acquired immunodeficiency syndrome
ALT: alanine aminotransferase
ART: antiretroviral therapy
AST: aspartate aminotransferase
CCR5: C-C motif chemokine receptor 5
CHMI: controlled human malaria infection
DV-2: Dengue virus serotype 2
EBOV: Ebola virus
GBV-C: GB virus type C
HAART: highly active antiretroviral therapy
HBV: hepatitis B virus
HCV: hepatitis C virus
HDV: hepatitis D virus
HEV: hepatitis E virus
HGV: hepatitis G virus
HIV: human immunodeficiency virus
HPgV: Human Pegivirus
IDU: intravenous drug use
IQR: interquartile range
MSM: men who have sex with men
NHL: non-Hodgkin lymphoma
NHL: non-Hodgkin lymphoma
TCR: T-cell receptor
TTV: Torque Teno virus
