# Supplementary material for: Human Pegivirus Type 1 Coinfections: Epidemiology, Heterogeneity and Clinical Implications
Source: J Gen Virol. 2026 Nov 5;107(11):002365. doi: 10.1099/jgv.0.002365 (PMC13739288; doi:10.1099/jgv.0.002365)
Supplement: Supplementary file 1 [file jgv-107-02365-s001.pdf]

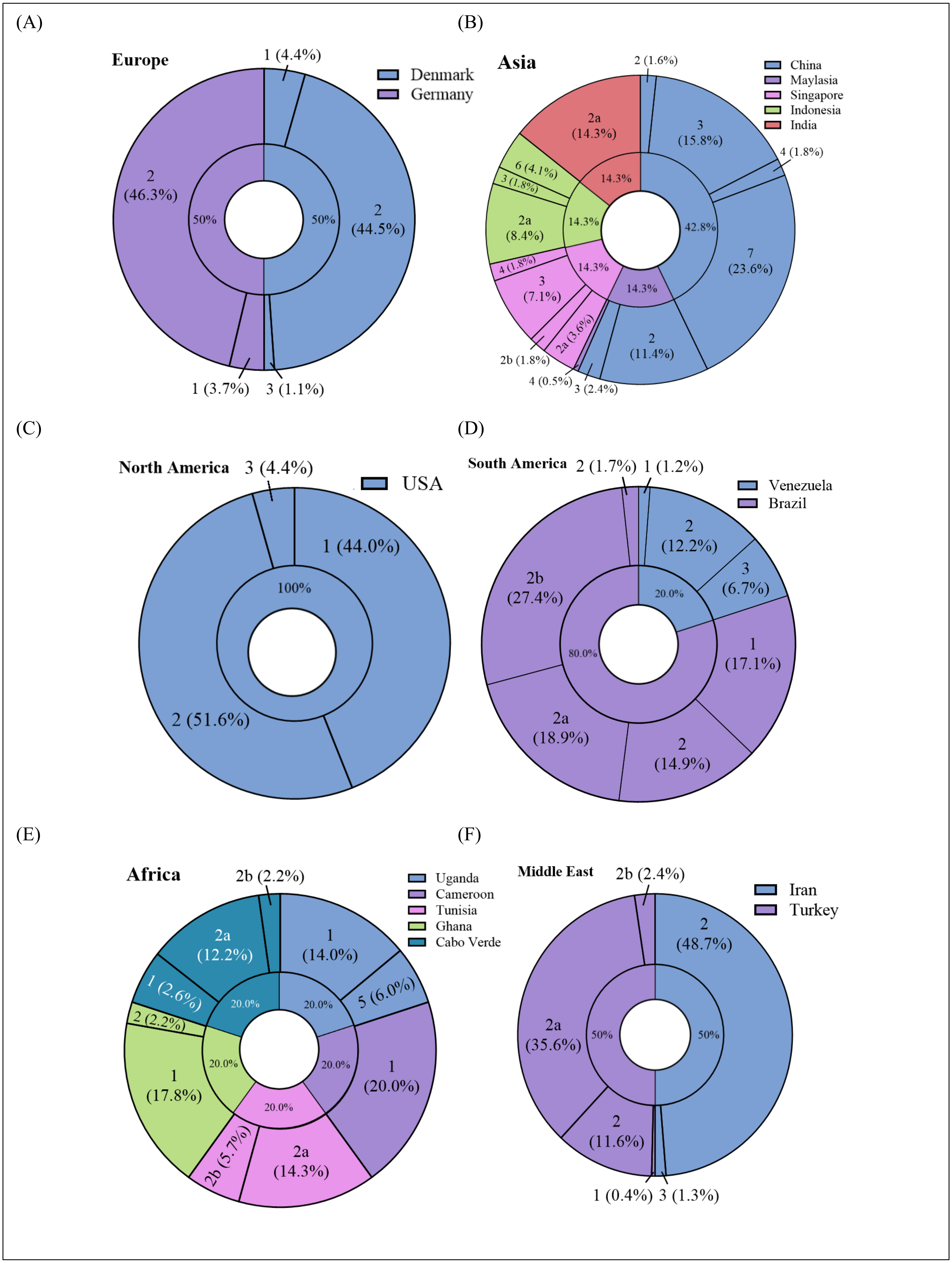

(A)
Europe
Denmark
Germany
1 (4.4%)
2 (44.5%)
50%
50%
2 (46.3%)
1 (3.7%)
3 (1.1%)
(B)
Asia
China
Maylasia
Singapore
Indonesia
India
2 (1.6%)
3 (15.8%)
4 (1.8%)
7 (23.6%)
42.8%
14.3%
2 (11.4%)
3 (2.4%)
4 (0.5%)
2a (3.6%)
2b (1.8%)
3 (7.1%)
4 (1.8%)
2a (8.4%)
3 (1.8%)
6 (4.1%)
2a (14.3%)
(C)
North America
USA
3 (4.4%)
1 (44.0%)
100%
2 (51.6%)
(D)
South America
Venezuela
Brazil
2 (1.7%)
1 (1.2%)
2 (12.2%)
3 (6.7%)
20.0%
80.0%
1 (17.1%)
2 (14.9%)
2a (18.9%)
2b (27.4%)
(E)
Africa
Uganda
Cameroon
Tunisia
Ghana
Cabo Verde
2b (2.2%)
1 (14.0%)
5 (6.0%)
20.0%
1 (20.0%)
2a (14.3%)
2b (5.7%)
1 (17.8%)
2 (2.2%)
1 (2.6%)
2a (12.2%)
(F)
Middle East
Iran
Turkey
2b (2.4%)
2 (48.7%)
50%
50%
2a (35.6%)
2 (11.6%)
1 (0.4%)
3 (1.3%)

***SI Figure 1*. *Breakdown of HPgV Genotype by Geographic Location* (%).** Note that some studies distinguished between genotypes 2a and 2b while some studies grouped them together under genotype 2. (A) **HPgV Genotype by Countries in Europe**. One study from Germany sequenced HPgV and identified genotypes 1, 2, and 3 (1) while one study from Denmark sequenced and identified genotypes 1 and 2 (2). The predominant genotype in Germany and Denmark was genotype 2 at 89.0% and 92.6%, respectively. (B) **HPgV Genotype by Countries in Asia.** Three studies from China found high frequencies of genotype 3 (36.8%) and 7 (55.1%) (3–5). A 2011 study identified a previously unrecognized HPgV genotype, now classified as genotype 7 (3). Following this discovery, the authors proposed a revised classification system, suggesting that genotypes 4, 6, and 7 be grouped together – based on phylogenetic analysis of partial and full-length genome sequences – reducing the total number of distinct HPgV genotypes to five though this new nomenclature has not been used in more recent studies. In Maylasia, Singapore, Indonesia, and India, one study from each country reported HPgV genotype data, identifying genotypes 2, 3, 2a, and 2a, respectively, as the most prevalent (6–9). This pattern contrasts with findings from China, where genotype 2 was the least common across three independent studies. These discrepancies challenge the notion of a clear geographic pattern in HPgV genotype distribution within Asia. (C) **HPgV Genotype by Countries in North America.** Genotype data were available from one study in the U.S. (10). In the U.S. cohort, genotypes 1 and 2 were almost evenly distributed, accounting for 44.3% and 51.4% of cases, respectively. D) **HPgV Genotype by Countries in South America.** Genotype data were available from one study in Venezuela (11) and four studies in Brazil (12–15). In Venezuela, genotype 2 was identified in 61% of co-infected patients, while in Brazil, genotype 2 was predominant, more specifically genotype 2b, accounting for 34.3% of all cases. (E) **HPgV Genotype by Countries in Africa.** Genotype data were available from single studies in Uganda, Cameroon, Tunisia, Ghana, and Cabo Verde (16–20). Uganda identified genotype 5 in 70% of patients while Ghana and Cameroon reported a predominance of genotype 1 in co-infected patients. In contrast, genotype 2a was most prevalent in Cabo Verde (61%) and Tunisia (71%). (F) **HPgV Genotype by Countries in the Middle East.** Genotype data were available from four studies, conducted in Iran and Turkey (21–24), where genotype 2 was most common in Iran with a prevalence of 97.4% while the predominant genotype in Turkey was 2a at a rate of 71.2% among co-infected patients.

1. Schwarze-Zander C, Blackard JT, Zheng H, Addo MM, Lin W, Robbins GK, et al. GB virus C (GBV-C) infection in hepatitis C virus (HCV)/HIV-coinfected patients receiving HCV treatment: importance of the GBV-C genotype. J Infect Dis. 2006 Aug 15;194(4):410–9.

2. Fahnøe U, Madsen LW, Christensen PB, Sølund CS, Mollerup S, Pinholt M, et al. Effect of direct-acting antivirals on the titers of human pegivirus 1 during treatment of chronic hepatitis C patients. Microbiol Spectr. 2024 Sep 3;12(9):e0064124.

3. Feng Y, Zhao W, Feng Y, Dai J, Li Z, Zhang X, et al. A novel genotype of GB virus C: its identification and predominance among injecting drug users in Yunnan, China. PLoS One. 2011;6(10):e21151.

4. Miao Z, Gao L, Song Y, Yang M, Zhang M, Lou J, et al. Prevalence and Clinical Impact of Human Pegivirus-1 Infection in HIV-1-Infected Individuals in Yunnan, China. Viruses. 2017 Feb 15;9(2):28.

5. Liu Z, Li L, Chen Z, Xu M, Zhang T, Jiao Y, et al. Prevalence of GB virus type C viraemia in MSM with or without HIV-1 infection in Beijing, China. Epidemiol Infect. 2012 Dec;140(12):2199–209.

6. Ng KT, Takebe Y, Chook JB, Chow WZ, Chan KG, Abed Al-Darraji HA, et al. Co-infections and transmission networks of HCV, HIV-1 and HPgV among people who inject drugs. Sci Rep. 2015 Oct 13;5:15198.

7. Lee CK, Tang JWT, Chiu L, Loh TP, Olszyna D, Chew N, et al. Epidemiology of GB virus type C among patients infected with HIV in Singapore. J Med Virol. 2014 May;86(5):737–44.

8. Anggorowati N, Yano Y, Subronto YW, Utsumi T, Heriyanto DS, Mulya DP, et al. GB virus C infection in Indonesian HIV-positive patients. Microbiol Immunol. 2013 Apr;57(4):298–308.

9. Alex D, Steve RJ, Ramalingam VV, Demosthenes JP, Vijayalakshmi G, Ghale BC, et al. Prevalence and potential impact of human pegivirus-1 on HIV-1 disease progression among Indian PLHIV. J Infect Dev Ctries. 2024 Jul 29;18(7):1108–17.

10. Blackard JT, Ma G, Welge JA, King CC, Taylor LE, Mayer KH, et al. GB Virus C (GBV-C) Infection in Hepatitis C Virus (HCV) Seropositive Women with or at Risk for HIV Infection. PLoS One. 2014;9(12):e114467.

11. Rodríguez AK, Garzaro DJ, Loureiro CL, Gutiérrez CR, Ameli G, Jaspe RC, et al. HIV-1 and GBV-C co-infection in Venezuela. J Infect Dev Ctries. 2014 Jul 14;8(7):863–8.

12. Alcalde R, Nishiya A, Casseb J, Inocêncio L, Fonseca LAM, Duarte AJS. Prevalence and distribution of the GBV-C/HGV among HIV-1-infected patients under anti-retroviral therapy. Virus Res. 2010 Aug;151(2):148–52.

13. Niama RF, Mayengue PI, Nsoukoula BCM, Koukouikila-Kossounda F, Badzi CN, Mandiangou AF, et al. Genetic variability of human pegivirus type 1 (HPgV-1) among Congolese co-infected with hepatitis C virus in Brazzaville, Congo. IJID Reg. 2023 Jun;7:191–2.

14. Nunes PF, da Cruz Coelho E, da Silva JR, da Silva Costa CC, Sampaio RMA, Frade PCR, et al. Hepatitis C and Human Pegivirus Coinfection in Patients with Chronic Hepatitis C from the Brazilian Amazon Region: Prevalence, Genotypes and Clinical Data. Viruses. 2023 Sep 7;15(9):1892.

15. Giret MTM, Miraglia JL, Sucupira MCA, Nishiya A, Levi JE, Diaz RS, et al. Prevalence, incidence density, and genotype distribution of GB virus C infection in a cohort of recently HIV-1-infected subjects in Sao Paulo, Brazil. PLoS One. 2011 Apr 5;6(4):e18407.

16. de Pina-Araujo IIM, Horta MA, do Amaral Mello FC, Soares CC. Human pegivirus 1 in Cabo Verde: prevalence and genotypic distribution among HIV-infected individuals. Arch Virol. 2021 May;166(5):1345–53.

17. N'Guessan KF, Boyce C, Kwara A, Archampong TNA, Lartey M, Sagoe KW, et al. Human pegivirus (HPgV) infection in Ghanaians co-infected with human immunodeficiency virus (HIV) and hepatitis B virus (HBV). Virus Genes. 2018 Jun;54(3):361–7.

18. Yirrell DL, Wright E, Shafer LA, Campbell E, Van der Paal L, Kaleebu P, et al. Association between active GB virus-C (hepatitis G) infection and HIV-1 disease in Uganda. Int J STD AIDS. 2007 Apr;18(4):244–9.

19. Ben Dhifallah I, Ayouni K, Chouiha A, Sadraoui A, Hogga N, Hammami W, et al. Genotype Distribution and Prevalence of Human Pegivirus among High-Risk Populations in Tunisia. Intervirology. 2016;59(3):170–8.

20. Luk KC, Berg MG, Naccache SN, Kabre B, Federman S, Mbanya D, et al. Utility of Metagenomic Next-Generation Sequencing for Characterization of HIV and Human Pegivirus Diversity. PLoS One. 2015;10(11):e0141723.

21. Yazdani L, Ravanshad M, Khanlari Z, Dawood Mousavi Nasab S, Ali Ahmadi N, Imanzad M. Prevalence of GBV-C among Iranian HBV positive patients using PCR-RFLP technique. Gastroenterol Hepatol Bed Bench. 2013;6(Suppl 1):S70-76.

22. Ghanbari R, Ravanshad M, Hosseini SY, Yaghobi R, Shahzamani K. Genotyping and infection rate of GBV-C among Iranian HCV- infected patients. Hepat Mon. 2010;10(2):80–7.

23. Köksal MO, Pirkl M, Sarsar K, Ilktaç M, Horemheb-Rubio G, Yaman M, et al. Interplay between HIV and Human Pegivirus (HPgV) Load in Co-Infected Patients: Insights from Prevalence and Genotype Analysis. Viruses. 2023 Dec 19;16(1):5.

24. Yücebağ-Duranay E, Kocazeybek BS, Kuşkucu MA. Frequency and Genotype Diversity of Human Pegiviruses (HPgV) in People Infected with Human Immunodeficiency Virus (HIV). Infect Dis Clin Microbiol. 2025 Mar;6(3):77–87.
